# Implementation of a 2D Wavelet Method to Probe Mixed Layer Height Using Lidar Observations

**DOI:** 10.3390/ijerph16142516

**Published:** 2019-07-14

**Authors:** Ning Zhang, Fuyan Yang, Yan Chen

**Affiliations:** 1 School of Atmospheric Sciences, Nanjing University, Nanjing 210023, China; 2 Guizhou Institute of Mountain Environment and Climate, Guiyang 550002, China; 3 Jiangsu Climate Center, Nanjing 210009, China

**Keywords:** mixed layer height, 2D wavelet, lidar observations

## Abstract

A new method was developed to estimate mixed layer (ML) height with light detection and ranging (lidar) observations using a 2Dimensional (2D) wavelet method, which can consider the diurnal variation characteristics of ML height. Ideal signals and real lidar observations in Shanghai, China were used to evaluate the new method. The results showed that the new method is insensitive to the type of wavelet filters. The estimated ML heights obtained by the 2D wavelet method agreed well with both of the previous methods developed for the ML height probing using lidar, including the gradient method, the 1D-wavelet method, the standard deviation method, and the conventional radiosonde method. The primary differences among the results obtained via the different lidar methods occurred in the early morning or later afternoon; when the ML is well mixed, very small differences were observed among the different lidar methods. The new method showed better determination skills than other methods when compared to the radiosonde observation results. It also performed well when there were missing profiles or observation errors and it made the new method suitable for operations where data quality control may be missed.

## 1. Introduction

The planetary boundary layer (PBL) height is an important parameter that helps to characterize the PBL. The dispersion of air pollutants is largely dependent on PBL height and its diurnal variation. PBL height determines the height to which an air pollutant can reach via diffusion [1,2,3], and it is an important parameter for predicting pollution. Moreover, the dynamics of the PBL and the concentration of ground pollutants mutually affect each other [4,5,6]. Determining PBL height is also one of the key steps of the turbulence closure scheme in numerical models [7]. For these reasons, estimating PBL height accurately is of great significance in the study of physical processes in the PBL and its parameterization for use in numerical simulations.

Conventional meteorological sounding is the most widely used method for estimating PBL height [8,9,10,11,12,13]; however, the traditional meteorological sounding method has a low probing frequency (usually twice a day) and thus does not capture the continuous of changes in PBL height. Especially in eastern China, operational observations are carried at 08:00 and 20:00 local time, the observations at 20:00 may keep the daytime boundary layer information in the residual layer, but they fail to get the detailed daytime information of boundary layer structure.

With the rapid development of remote sensing technology, ground-based remote sensing methods have been implemented to probe the PBL and have become common methods to estimate PBL height. The remote sensing method termed light detection and ranging (lidar) is characterized by its small volume, low weight, continuous observation, and high resolution [10]. In recent years, lidar observations have been widely used in determining PBL height, especially the PBL height in the daytime [11,12,13,14,15,16,17,18], which is also termed mixed layer (ML) because of the strong vertical turbulence mixing in the daytime. Haeffelin et al. [19] used one-month automatic profiling lidar and ceilometer observations from the Integrated Carbon Observing System to compare five retrieval methods, and found that current methods have no evidently different skill in detecting one or multiple significant aerosol gradients from lidar and ceilometer backscattering signals. This study also stressed the importance of pre-processing to reach sufficient signal-to-noise ratios.

Probing ML height using lidar signals is most commonly based on the hypothesis that the ML is well-mixed and the inversion layer at the top of the ML restrains the vertical transport of aerosol particles and water vapor from the ML to the free atmosphere. This results in the extinction ability of the ML atmosphere being greater than that of free atmosphere above. ML height is determined by finding the height at which an abrupt change occurs in the vertical profile of the backscattered signals or extinct coefficients. Some common methods that probe ML height in conjunction with lidar include the gradient (GRAD) method [20], the 1D-wavelet (WH) method [21], and the standard deviation (STD) method [19].

The GRAD method determines ML height directly according to the decay rate of lidar signals with height, and the height at which the maximum of the first or second derivative of the signal appears is taken as the ML height. In the present study, the height at which the maximum negative gradient of the probing signal occurs is defined as the ML height. The GRAD method is simple and convenient, but it is susceptible to environmental noise, leading to inaccuracies in the ML height determined [18].

There is always strong entrainment at the interface of the free atmosphere and the ML [20], which is reflected in the lidar backscatter signals as dramatic signal changes at the top of the ML. Hence, the height with the maximum standard deviation of the signal is defined as the ML height. The STD method is resistant to disturbance from weak turbulence, but it is inapplicable in the case of weak capping inversion.

The 1D wavelet covariance transform defined by Gamage and Hagelberg [21] is used to detect signal transitions. Usually, the Haar wavelet is selected as the wavelet basis function. Larger values of the wavelet covariance function suggest stronger similarity between the signal function and the Haar function, leading to a larger step change. Therefore, the height at which the wavelet covariance function obtains the maximum value is defined as the ML height in the WH. 

Currently, lidar observations are widely deployed in air quality research and weather services in China, because of their high-spatiotemporal resolutions. Most current operations running in China lack proper data preprocessing and sometimes the instruments are not well maintained. A robust and stable method is needed in the real operations. The current existing methods that extract the ML height from lidar data only consider the instantaneous lidar signal change with height. These methods can output reasonable diurnal variations of ML with proper pre-processing and data quality control, but may fail with poor quality observations. In the present study, we propose using a 2D wavelet method to determine ML height based on the principle of image edge detection. The results were compared with those obtained via the existing methods to discuss the viability of estimating ML height accurately using the 2D wavelet method introduced herein. This paper includes four sections: (1) Introduction; (2) The New Method based on 2D Wavelet Analysis; (3) Data and Results and (4) Conclusions.

## 2. The New Method Based on 2D Wavelet Analysis

Analysis of probing signals using previous methods has focused on single instantaneous vertical observation profiles with the aim of finding the “abrupt change point” of the signal, and little attention has been paid to the diurnal variations of the lidar signals. However, the actual ML height shows very typical diurnal variation characteristics. If the diurnal variation of the lidar signal profiles is considered as a 2D image, the determination of ML height involves locating the edge between the scattering signal of free air and the atmospheric scattering signal of the ML in the 2D image, namely an “abrupt change line”, which would signify the diurnal variation patterns.

The method used to extract the image edge in this study is an edge detection skill based on 2D wavelet decomposition of a 2D lidar signal image. The components of wavelet decomposition are then used to detect the image edge. The principal idea of this method is to discard low frequency, unrefined images and reconstruct them with high-frequency detail at different scales, thereby extracting the image edge. The image undergoes wavelet decomposition to obtain both low- and high-frequency signals. The low-frequency signal reflects the basic profile of the image, whereas the high-frequency signal corresponds to the detailed image information.

After wavelet decomposition is performed on an image, the low-frequency portion *a_J_* and the high-frequency portion *d_J_* are obtained by smoothing filters. The image edge is often present in the high-frequency region of the image after filtering. Thus, the image edge can be extracted by reconstructing the high-frequency portion *d_J_*.

Suppose that the maximum decomposition layer of the image is J; the rough image obtained by wavelet decomposition in layer J (i.e., *a_J_*) is then discarded. Three high-frequency detail images (i.e., $d_{J}^{1}$, $d_{J}^{2}$, and $d_{J}^{3}$ in layer J) are then used for reconstruction as follows:

(1) $$a_{J-1}=d_{J}^{1}+d_{J}^{2}+d_{J}^{3}$$

Next, $a_{J-2}$ is taken as the rough image $a_{J-1}$ obtained by wavelet decomposition in layer *J* − 1, and Equation (2) is used for the reconstruction:

(2) $$a_{J-2}=a_{J-1}+d_{J-1}^{1}+d_{J-1}^{2}+d_{J-1}^{3}$$

Similarly, Equation (2) can be used for reconstruction to obtain $a_{J-3}$, and so on, until $a_{0}$ is obtained.

When extracting ML height based on the 2D wavelet method, the height corresponding to the maximum value of the reconstructed image based on the detailed image signal after 2D wavelet transform is defined as the ML height. Hereafter, this approach is referred to as the 2D wavelet (W2D) method. In the transform progress, the threshold between the low and high frequency depends on the image characteristics, and it may be different in the vertical (spatial) and the horizontal (temporal) direction. In this study, two-layer decomposition was used, the codes were programed with MATLAB, and the default parameters in the 2D wavelet filters were used. Given the spatial and vertical observation resolution discussed in Section 3.1, the thresholds between the low and high frequency were 0.5 min^−1^ in the horizontal direction and the filter scale in the vertical direction was about 60 m depending on the signal qualities.

## 3. Data and Results

### 3.1. Influence of Wavelet Filter Selection on the W2D Estimation Results

The observational data used in this study were obtained using a micro-pulse lidar (MPL-4B) produced by the SigmaSpace Corporation. The observation site was located at the Shanghai Center for Urban Environmental Meteorology (31°13′ N, 121°32′ E), and the instrument was installed on the roof of the main building of the center. The extinction coefficients calculated with the normalized relative backscatter (NRB) signals were used for the ML height estimation in this paper. The extinct coefficient was defined as $c=\frac{dI_{\lambda }}{I_{\lambda }\mathrm{dx}}$, *I_λ_* is the incoming radiation intensity at the spectra λ, and x is the transport distance. The unit of extinct coefficient is m^−1^, and we used 100 m^−1^ in this paper, hereafter hm^−1^. The spatiotemporal distribution of the normalized backscattering signals can, to some extent, reflect the vertical distribution characteristics of aerosols and the ML structure; meanwhile, it can be used to calculate the aerosol extinction coefficient profiles and the range correction signals. The data had a time resolution of 30 s and a vertical resolution of 30 m. Sunny day observations in 2012 and 2013 were used, including 20 sunny days in the summer of 2012, 25 sunny days in the summer of 2013, and 35 sunny days in November and December of 2012 and 2013.

In 1-dimensional (1-D) wavelet transform, the selection of the wavelet basis functions has a significant effect on the results. When the WH method was used to estimate ML height, the Haar wavelet transform was selected because it can reflect the abrupt changes of lidar signals at the top of the ML. To investigate the effect of different wavelet filters on the results of the ML height extracted by the W2D method, we compared four wavelet filters: the Daubechies wavelet (db2), the Coiflets wavelet (coif3), the symmetric wavelet (sym2), and the biorthogonal wavelet (bior3.7).

**Figure 1:**
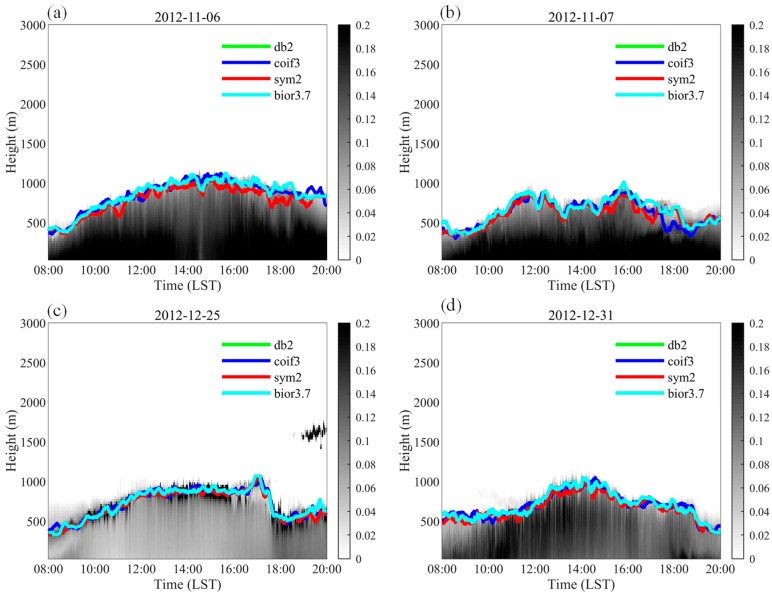
The diurnal variations of mixed layer (ML) heights (solid lines) calculated by the 2D wavelet method based on different wavelet filters: (**a**) 6 November; (**b**) 7 November; (**c**) 25 December and (**d**) 31 December 2012. (The grey shades show the extinct coefficients, unit: hm^−1^; and the effect of boundary layer cloud on the extinct coefficients were also observed in (**c**)). The influences of cumulus on the lidar observations is also shown in (**c**). The grey shades show the extinct coefficients, unit: hm^−1^.

**Figure 2:**
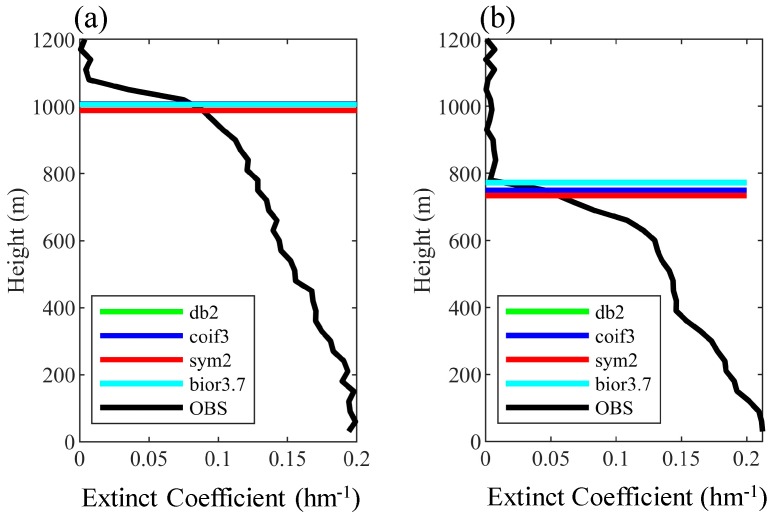
The vertical extinct coefficient profiles and ML height determined by the 2D wavelet method at (**a**) 14:00 and (**b**) 17:00 6 November 2012.

Figure 1 shows the 15-min averaged ML height detected based on the W2D method in Shanghai on 6 November, 7 November, 25 December, and 31 December 2012. The W2D method can adequately estimate the ML height and its diurnal variation. For these four days, the ML height ranged between 300 m and 1000 m, with the maximum value being observed in the afternoon. Figure 2 illustrates the vertical profiles of the extinct coefficient and the ML height determined by the W2D method using different wavelet filters. The results are very close. The 30 min mean, maximum and minimum of ML height in the summer sunny days in 2012 estimated by the four wavelet filters are also shown in Table 1. The statistical period was set to 10:00–18:00 LT (Local Time). Table 1 shows that the estimation results were generally consistent when the db2 and sym2 basis functions were used, which is logical because the sym2 wavelet is an improvement of the db2 wavelet. Moreover, in addition to the characteristics of the db wavelet filter, the wavelet basis function ψ(t) of sym2 was nearly symmetric. When the coif3 and bior3.7 basis functions were used, the means were 933 m and 935 m, respectively, whereas the minimum values were markedly different (433 m and 477 m, respectively). Regarding the means of the four methods, there was little difference in the results from using different wavelets. This indicates that the results from the W2D method were insensitive to the selection of wavelet filter. For this reason, only results from one filter (here, the bior3.7 wavelet filter) were selected for the W2D method to estimate ML height in the following discussion. Figure 2 also shows that that the aerosol layer is not that well-mixed with the extinct coefficient value decreases from the surface to upward; the greatest gradient occurred at the top of ML (about 1000 m at 14:00 and 800 m at 17:00), several secondary gradient peaks occurred below.

### 3.2. Comparisons of Different Methods Using Ideal Data

To compare the W2D methods with previous methods (the GRAD, WH and STD methods) and discuss the advantages of the W2D method, ideal diurnal variations of lidar observation profiles were created to test the performance of different methods. An ideal ML height diurnal variation was created using the following equations:

(3) $$h\left(t\right)=h_{0}+H_{r}sin(\frac{t-t_{0}}{T}\pi )+\alpha \left(t\right)H_{p}$$

Here *h*(*t*) is the ML height at time *t*, *h*_0_ is the ML height at *t*_0_ (*h*_0_ is set to 200 m), when the ML height begin to increase (here, *t*_0_ = 08:00 LT to be consistent with the real observation data); *H_r_* is the diurnal change range of ML height, it was set to 800 m and it means the ML height ranges between 200 m and 1000 m. *T* is the daytime variant periods, it was set to 12 h. *α*(*t*)*H_p_* is the perturbation term, *α*(*t*) is the scale factor, it is a random number between −0.5 and 0.5 and *H_p_* is height range of the perturbation. The extinct coefficients were to 50 (the value does not impact the analysis) below *h*(*t*) and 0 above it. Four sets of data with the temporal resolution of 30 s and vertical resolution of 30 m were calculated using Equation (3) and the 15 min averaged ML heights were compared to analyze the performance of different methods. In the Case 0 experiment, *H_p_* was set to 0 and the ML height showed a sinusodial change in 12 h (Figure 2a). 

**Figure 3:**
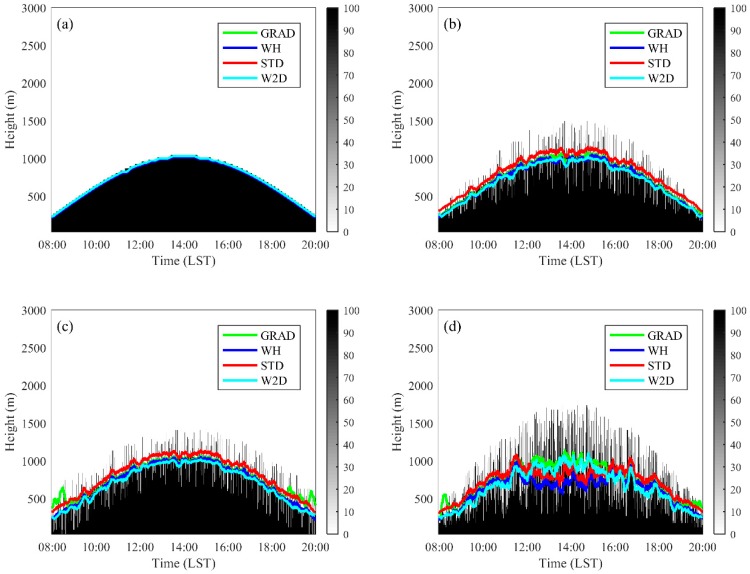
The ML heights estimated by different methods using ideal data: (**a**) the Case 0 experiment; (**b**) the Case 1 experiment; (**c**) the Case 2 experiment; and (**d**) the Case 3 experiment.

In the Case 0 experiment, all four methods could estimate the ideal ML height variation well and agreed with one another, as shown in Figure 3a. In the Case 1, Case 2 and Case 3 experiments, random perturbations were added to the signal at every time step. In the Case1 experiment, the *H_p_* was set to 0.5 *h*(*t*), *h*(*t*) is the ML height in the Case 0 experiment at the respective time; *H_p_* was set to a fixed valued of 250 m in the dieal2 experiment. In the Case 3 experiment *H_p_* was set as the *h*(*t*) in the ideal 0 experiment. The mean bias (the experiments minus the Case 0 experiment), correlation coefficient and root square mean error (RMSE) of estimated ML heights between the Case 1, Case 2, Case 3 experiment and the Case 0 experiment were used to evaluate the method performance, as shown in Table 2. The results show that in the Case 1 and Case 2 experiments, all four methods showed good correlation between the estimated results with the results of the Case 0 experiment, and the correlation coefficients were close to 1 (greater than 0.95 except the 0.94 of the STD method in the Case 2 experiment). All of the methods tended to underestimate the daytime averaged ML height in all experiments except the GRAD and STD methods, which overestimated them in the Case 2 experiment. The GRAD, WH and W2D methods showed similar performance with the bias, and RMSEs were close to or smaller than the vertical signal resolution (30 m) in the Case 1 and Case 2 experiments, while the STD method showed relative higher error level with the bias of 62.6 m and the RMSE of 68.5 m in the Case 3 experiment. In the Case 3 experiment, when the perturbation term appeared in a wider range, the W2D results had a high correlation with the Case 0 results, with an R value of 0.98, while the correlation coefficients of other three methods decreased dramatically. The GRAD method and STD method outputted small bias in the averaged ML height, but the GRAD method showed a lower correlation coefficient of 0.97 and the STD method underestimated the ML height in the middle period (from 12:00 to 16:00, Figure 3d). Two more series of experiments were designed with different function forms of *H_p_*. In the first one, the *H_p_* was set as a function of *h*(*t*), *H_p_* = *bh*(*t*); and in the second one, *H_p_* was set as a fixed value. The method evaluation results are shown in Table 3. It is not surprising that the performances of all methods decreased when the absolute value of *H_p_* increased. All methods tended to underestimate ML height, as shown in the tests of the Case 1–Case 3 experiments, except the STD method, which always output a positive bias. The GRAD and W2D method output lower biases and RMSEs than the WH and STD methods and the W2D method results have the highest correlation coefficient (0.98 when *b* = 0.8 and 0.99 in all other experiments). The bias and RMSE increase with hp in both the GRAD and W2D methods, because the profiles become more deviated from the well mixed condition. When the absolute value of *H_p_* is low, the absolute value of bias and RMSE output by the W2D method were greater than these of the GRAD method, but the increasing ratio of bias and RMSE of the W2D method was much smaller than those of the GRAD method, which indicated that the W2D method is more robust. As shown in Figure 2 and the following Section 3.3, well-mixed signal profiles are not always the case in the real observations.

### 3.3. Comparisons of Different Methods Using Real Data

**Figure 4:**
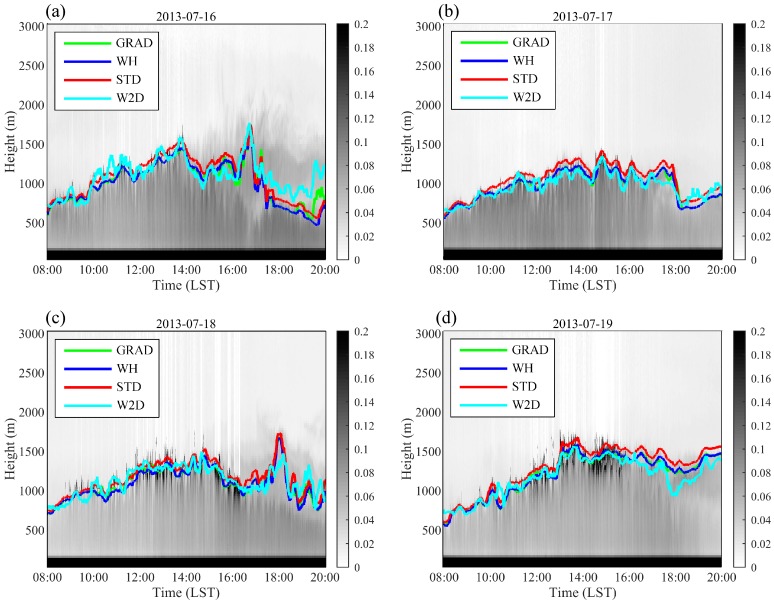
The diurnal variations of ML heights (solid lines) calculated by the 2D wavelet method based on different wavelet filters: (**a**) 16 July, (**b**) 17 July; (**c**) 18 July, (**d**) 19 July 2013. The grey shades show the extinct coefficients, unit: hm^−1^.

The estimated results of the W2D method using the real field observations in Shanghai were also compared to those of the GRAD, WH, and STD methods. Figure 3 and Figure 4 illustrate the diurnal variation in the measured ML height based on lidar for four sunny days in both the summer and winter of 2013. The ML height calculated by the W2D method and these calculated by the GRAD, WH, and STD methods delineated similar development and change processes of the ML. Overall, the ML height determined by the four methods was highly consistent. In general, the results obtained from the STD method were higher compared with the remaining methods. Little difference was found in the results for the WH and GRAD methods, whereas the results estimated by the W2D method were slightly lower in the mean values. The start time of ML development was later in winter than in summer, and the ML began to grow rapidly after 10:00 LT. On average, the ML height was lower in December than in July, peaking at approximately 1300 m. On July 16, the ML heights determined by the different methods were clearly distinct after 18:00 LT. On July 17, the ML height value between 17:00 LT and 18:00 LT estimated by the W2D method was small, and the ML height dissipated earlier than those estimated by the other three methods. These distinct results usually occured in the transit period of the PBL diurnal variations.

Table 4 and Table 5 list the mean, maximum, and minimum of daytime ML heights estimated under sunny conditions in summer and winter, respectively. In summer, the GRAD method showed little difference compared with the WH, with only a 2 m difference in the mean. The STD method produced higher mean and extreme values than the other three methods. The mean derived from the W2D method was higher than those of the GRAD and WH methods, but lower than that of the STD method. There was little difference in the results of the W2D method compared with those of the GRAD and WH methods. Regarding the mean of the estimation results in winter, the results of the W2D and STD methods were close, with means of 961 m and 1007 m, respectively. In winter, the ML height extracted by the W2D method was relatively close to the STD method result, but their maximum values showed a large difference.

According to Table 4 and Table 5, the seasonal difference in the mean of the ML height ranged from 200 m to 300 m, and the difference in the maximum of the ML height reached 300 m or higher during the sunny daytimes in winter and summer. This is because the sunrise is earlier and the sunshine duration is longer in summer and because the ML begins to develop earlier in summer than in winter. In winter, the ML began to enter the rapid development stage only at approximately 10:00 LT. Taking the W2D method results as an example, the ML had a maximum height of 1382 m, a mean height of 961 m, and a minimum height of 405 m in winter; in summer, the ML had a maximum height of 2016 m, a mean height of 1170 m, and a minimum height of 697 m. Between the two seasons, the difference in the minimum was 292 m, while the difference in the maximum reached 634 m. 

Table 6 and Table 7 are the inter-comparisons of the estimation results among different methods. All of the correlation coefficients were greater than 0.75 in both winter and summer, which means all method had good agreement. The correlation coefficients in summer were higher than those in winter, because the daytime boundary layer has strong turbulence mixing and is closer to well-mixed in summer due to the strong indent solar radiation and warmer land surface.

**Figure 5:**
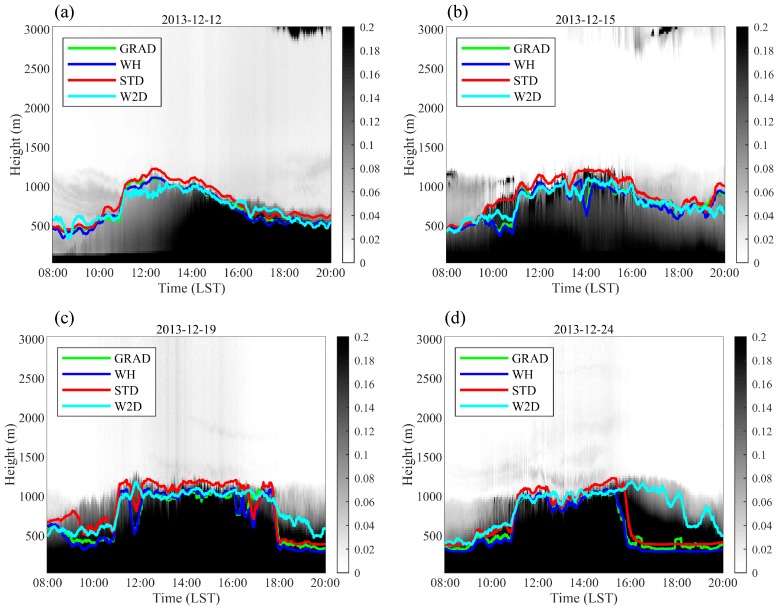
The diurnal variations of ML heights (solid lines) calculated by the 2D wavelet method based on different wavelet filters: (**a**) 12 December, (**b**) 15 December, (**c**) 19 December, and (**d**) 24 December 2013. The influences of cumulus on the lidar observations is also shown in (**c**). The grey shades show the extinct coefficients, unit: hm^−1^.

**Figure 6:**
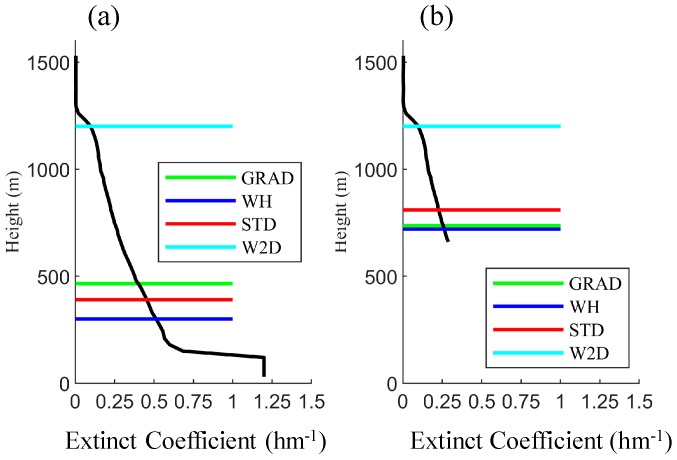
Vertical profiles and the ML heights calculated by different methods at 16:30 20 December 2013: (**a**) using the original data; (**b**) using the data after quality control. The black solid line is the vertical profile of extinct coefficients.

Figure 5d also shows how the data quality impacted on different methods. The observed ML started to develop at 10:00 and reached above 1000 m at 14:00. Before 16:00 the ML heights calculated by different methods were similar, but after 16:00 the ML heights calculated by the GRAD, WH and STD methods dropped dramatically to less than 500 m. The W2D method showed an ML which decreased gradually after 16:00. The reason for this is that strong anomalies appeared in the observations at the lowest part of the PBL (less than 300 m) as in Figure 6, and the extinct coefficient greater than 1.0 hm^−1^. This leads to great gradients at the bottom of PBL and caused the dramatic decreasing in the GRAD, WH and STD methods. Another gradient peak also appeared at the top of ML with a height of 1200 m; the W2D method kept the information from the previous time, so it could still keep a high ML height. After a simple data-quality control (removing the values greater than 0.25 hm^−1^), the GRAD, WH and STD methods outputted higher ML height but yet much lower than the results of the W2D method. As discussed in Section 3.1, this also proves the robustness of the W2D method.

### 3.4. Comparisons with the Conventional Radiosonde Method

**Figure 7:**
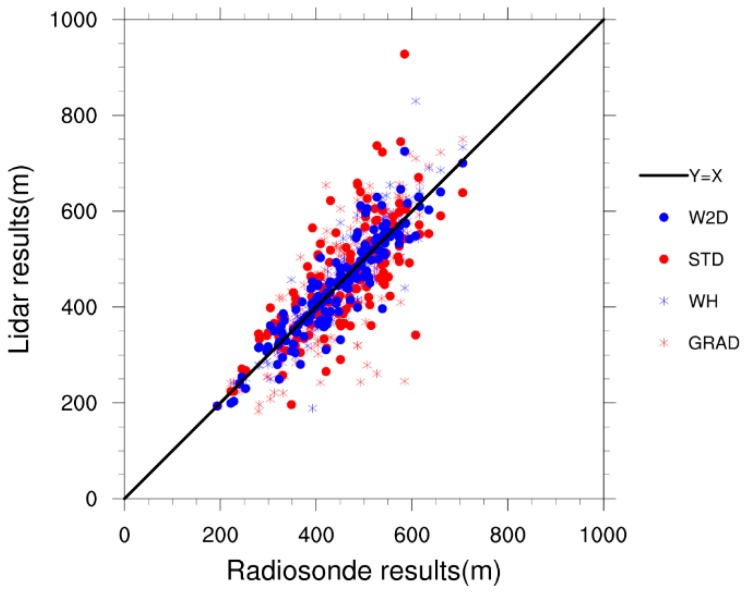
Comparisons of different lidar methods with the radiosonde observations, the black line is the reference line of Y = X.

The ML heights at 08:00 LT and 20:00 LT of the 80 days mentioned in Section 3.1 were estimated using different lidar methods and compared to the results calculated with the conventional radiosonde method. Eight sets of data were not used because at least one method outputted unreasonable results. The 152 sets of data were compared, and the results were shown in Figure 7 and Table 8. The parcel method was used for the conventional radiosonde observations, because the bulk Richardson number method failed frequently due to the low vertical resolution of the profiles and the accuracy of the wind observations at the respective time, and another conventional method, the temperature gradient method, strongly depends on a manually selected threshold value. The parcel method assumes an air parcel is lifted following the dry adiabat starting with the measured or expected maximum potential temperature up to its intersection with the potential temperature profile. the intersection height was taken as the ML height. The outputs of the parcel method are close to these of the bulk Richardson number method according to previous studies [22].

The results showed that the W2D method had a better agreement with the radiosonde results than other methods, with the smallest bias of 2.6 m and an RMSE of 40.6 m, and the highest correlation coefficient of 0.90. The STD method overestimated the ML heights to the same degree as the W2D method but output a much higher RMSE, and the lowest correlation coefficient of 0.72. The GRAD method and the WH method tended to underestimate the ML height, the bias of the GRAD method was −3.0 m, the RMSE was 80.5 m and the R was 0.79. The WH methods also showed a relatively good performance, with an RMSE of 47.2 m and the correlation coefficient of 0.85.

## 4. Conclusions

This study proposes the use of the 2D wavelet (W2D) method, which is based on the principle of image edge detection with lidar retrieval data (normalized signal intensity and extinction coefficient) to estimate the ML height. Both ideal signals and real lidar observations in Shanghai, China were used to test the new method. The ML heights probed by radiosonde observations and those determined by different lidar methods were compared, and the feasibility of using the method to estimate the ML height was verified. The db2, coif3, sym2, and bior3.7 wavelets were used to investigate the sensitivity of the W2D to the selection of wavelet functions. Overall, the W2D method was found to be insensitive to the selection of wavelet function. A series of ideal signals were used to test the new method, and the results showed that the W2D method is more robust than previous methods. 

The ML heights extracted from lidar data using the W2D and existing methods were compared using the real lidar observation signals. The ML height detected by the W2D method was relatively consistent with those detected by the GRAD, WH, and STD methods, clearly showing the evolution process of the ML. In summer, the result extracted by the W2D method was more consistent with the WH method result. In winter, the result extracted by the W2D method was close to that of the STD method because of the effect of a weak image edge in the ML. 

The estimation results from different lidar methods were also compared to the results determined by the radiosonde observations, and this also proves that the W2D method works better than other methods with lower error biases and RMSEs and higher correlation coefficients. This shows that the W2D method is suitable for operational applications with its high ability to endure more poor-quality observations such as missing data and high noise-signal ratios.

## Figures and Tables

**Table 1:** Daily mean, daily maximum, and daily minimum of 15-min average planetary boundary layer heights (m) from 10:00 to 18:00 LT.

Wavelet Filter Name	Mean	Maximum	Minimum
db2	903	1561	376
coif3	933	1547	433
sym2	903	1561	377
bior3.7	935	1531	477

**Table 2:** Evaluations of different methods using ideal data.

Method	Case 1			Case 2			Case 3		
	Bias (m)	R	RMSE	Bias	R	RMSE (m)	Bias	R	RMSE (m)
GRAD	−10.1	0.99	30.5	10.9	0.98	55.5	−12.5	0.97	60.2
WH	−17.9	0.99	37.5	−30.6	0.97	71.6	−151.0	0.79	213.9
STD	−4.0	0.99	29.3	62.6	0.94	68.5	−43.8	0.88	137.4
W2D	−31.5	0.99	41.4	−17.3	0.99	28.5	−50.3	0.98	71.8

All correlation coefficients passed the 95% significance level and all the difference passed the 5% significance level using the *t*-test method.

**Table 3:** Method evaluations with different Hp as *H_p_* = *bh*(*t*).

*b*		*Hp* = *bh*(*t*)			W2D	*Hp* (*m*)		*H_p_* as a Fixed Value			W2D
		GRAD	WH	STD				GRAD	WH	STD	
0.2	Bias	−2.5	−28.4	61.3	−15.3	50	Bias	−3.5	−29.1	59.4	−23.4
	R	0.99	0.97	0.99	0.99		R	0.99	0.97	0.99	0.99
	RMSE	12.2	65.5	66.9	24.3		RMSE	10.4	70.1	64.1	44.4
0.4	Bias	−6.4	−41.2	55.7	−22.84	100	Bias	−2.4	−27.1	59.0	−17.4
	R	0.99	0.97	0.99	0.99		R	0.99	0.98	0.99	0.99
	RMSE	22.6	75.1	64.2	35.5		RMSE	14.5	55.6	64.0	28.1
0.6	Bias	−8.0	−70.0	24.0	−36.4	150	Bias	1.3	−29.1	60.8	−15.2
	R	0.99	0.91	0.95	0.99		R	0.99	0.97	0.99	0.99
	RMSE	36.6	130.7	75.1	47.3		RMSE	23.9	64.5	66.3	20.6
0.8	Bias	−13.6	−128.8	−19.9	−42.8	200	Bias	7.2	−32.2	63.7	−14.4
	R	0.97	0.85	0.91	0.98		R	0.98	0.95	0.99	0.99
	RMSE	56.9	183.9	105.9	59.9		RMSE	38.2	86.0	68.9	23.8

All correlation coefficients passed the 95% significance level and all the difference passed the 5% significance level using the *t*-test method.

**Table 4:** Comparison of the ML height (m) determined by different methods for summer sunny days of 2013.

Method	Mean	Maximum	Minimum
GRAD	1147	2053	636
WH	1145	2046	644
STD	1231	2138	731
W2D	1170	2016	697

All correlation coefficients passed the 95% significance level and all the difference passed the 5% significance level using the *t*-test method.

**Table 5:** Comparison of ML layer height (m) estimated by different methods for winter sunny days of 2013.

Method	Mean	Maximum	Minimum
GRAD	917	1462	335
WH	910	1460	300
STD	1007	1550	390
W2D	961	1382	405

All correlation coefficients passed the 95% significance level and all the difference passed the 5% significance level using the *t*-test method.

**Table 6:** Inter-comparison of the ML heights (m) determined by different methods for summer sunny days of 2013.

Method	GRAD	WH	STD	W2D
GRAD	1.00	0.84	0.83	0.89
WH	0.84	1.00	0.82	0.78
STD	0.83	0.82	1.00	0.84
W2D	0.89	0.78	0.84	1.00

All correlation coefficients passed the 95% significance level and all the difference passed the 5% significance level using the *t*-test method.

**Table 7:** Inter-comparison of the ML heights (m) determined by different methods for winter sunny days of 2013.

Method	GRAD	WH	STD	W2D
GRAD	1.00	0.80	0.79	0.81
WH	0.80	1.00	0.77	0.76
STD	0.79	0.77	1.00	0.75
W2D	0.81	0.76	0.75	1.00

All correlation coefficients passed the 95% significance level and all the difference passed the 5% significance level using the *t*-test method.

**Table 8:** Comparison of the ML height (m) determined by different methods with the radiosonde results.

Method	Bias (m)	RMSE (m)	R
GRAD	−3.0	80.5	0.79
WH	−5.1	47.2	0.85
STD	5.4	91.6	0.72
W2D	2.6	40.6	0.90

All correlation coefficients passed the 95% significance level and all the difference passed the 5% significance level using the *t*-test method.
